# HSV-1 infection induces phosphorylated tau propagation among neurons via extracellular vesicles

**DOI:** 10.1128/mbio.01522-24

**Published:** 2024-08-27

**Authors:** V. Protto, M. T. Miteva, F. Iannuzzi, M. E. Marcocci, D. D. Li Puma, R. Piacentini, M. Belli, L. Sansone, A. Pietrantoni, C. Grassi, A. T. Palamara, G. De Chiara

**Affiliations:** 1 Department of Infectious Diseases, Istituto Superiore di Sanità, Rome, Italy; 2 Institute of Translational Pharmacology, CNR, Rome, Italy; 3 Department of Public Health and Infectious Diseases, Sapienza University of Rome, Laboratory affiliated to Istituto Pasteur Italia-Fondazione Cenci Bolognetti, Rome, Italy; 4 Department of Neuroscience, Università Cattolica del Sacro Cuore, Rome, Italy; 5 Fondazione Policlinico Universitario A. Gemelli IRCCS, Rome, Italy; 6 Department of Human Sciences and Promotion of the Quality of Life, San Raffaele Roma Open University, Rome, Italy; 7 Laboratory of Molecular, Cellular and Ultrastructural Pathology, IRCCS San Raffaele Roma, Rome, Italy; University of Pennsylvania, Philadelphia, Pennsylvania, USA; University of Aarhus, Aarhus, Denmark

**Keywords:** herpes simplex virus, extracellular vesicles, tau, Alzheimer’s disease

## Abstract

Extracellular vesicles (EV), key players in cell-to-cell communication, may contribute to disease propagation in several neurodegenerative diseases, including Alzheimer’s disease (AD), by favoring the dissemination of neurotoxic proteins within the brain. Interestingly, growing evidence supports the role of herpes simplex virus type 1 (HSV-1) infection in the pathogenesis of AD. Here, we investigated whether HSV-1 infection could promote the spread of phosphorylated tau (ptau) among neurons via EV. We analyzed the ptau species that were secreted via EV following HSV-1 infection in neuroblastoma cells and primary neurons, focusing particularly on T205, T181, and T217, the phosphorylation sites mainly associated with AD. Moreover, by overexpressing human tau tagged with GFP (htau^GFP^), we found that recipient tau knockout (KO) neurons uptook EV that are loaded with HSV-1-induced phtau^GFP^. Finally, we exploited an *in vivo* model of acute infection and assessed that cerebral HSV-1 infection promotes the release of ptau via EV in the brain of infected mice. Overall, our data suggest that, following HSV-1 infection, EV play a role in tau spreading within the brain, thus contributing to neurodegeneration.

**IMPORTANCE:**

Herpes simplex virus type 1 (HSV-1) infection that reaches the brain has been repeatedly linked with the appearance of the pathognomonic markers of Alzheimer’s disease (AD), including accumulation of amyloid beta and hyperphosphorylated tau proteins, and cognitive deficits. AD is a multifactorial neurodegenerative disease representing the most common form of dementia in the elderly, and no cure is currently available, thus prompting additional investigation on potential risk factors and pathological mechanisms. Here, we demonstrate that the virus exploits the extracellular vesicles (EV) to disseminate phosphorylated tau (ptau) among brain cells. Importantly, we provide evidence that the HSV-1-induced EV-bearing ptau can be undertaken by recipient neurons, thus likely contributing to misfolding and aggregation of native tau, as reported for other AD models. Hence, our data highlight a novel mechanism exploited by HSV-1 to propagate tau-related damage in the brain.

## INTRODUCTION

Extracellular vesicles (EV) are cell-secreted membrane vesicles involved in cell-to-cell communication. EV are highly variable in size and biogenesis as they can either originate from the pinching off of the plasma membrane [microvesicles (MV) usually ranging from 50 to 1,000 nm)] or being formed inside multivesicular bodies and released after their fusion with the plasma membrane [exosomes (exo) with a diameter ranging from 30 to 150 nm] (1). Both MV and exo carry proteins and several RNA subtypes that can trigger various physiological effects when delivered to the recipient cells. Despite their heterogeneity, EV share the presence of molecules such as CD63, CD9, CD81, alix, and TSG101, but the composition and distribution of these surface molecules vary greatly depending on the tissue of origin. Additional complexity results within virus infections, since viruses may deeply affect EV composition (2–4). Indeed, viruses can hijack EV pathways to benefit many steps of their life cycle, including egress from the infected cells and immune escape strategies (5, 6). On the other side, EV can contain miRNAs or other antiviral molecules that can alert surrounding cells and stimulate antiviral responses (7, 8). The interplay between viruses and EV is even more intricate considering that those components of cellular trafficking known to be exploited by viruses for building up replicative centers are also involved in the EV biogenesis (1).

Herpes simplex virus type 1 (HSV-1), a neurotropic virus able to establish life-long latent infection in humans with periodic reactivation, has been shown to modulate EV content in many ways. Previous studies showed that HSV-1 infection stimulates the release of EV from cells (7), and recent findings highlight that HSV-1 can hijack EV pathways, hiding virions inside MV both to infect non-permissive cells and to shield the virus from neutralizing antibodies (5). Conversely, HSV-1-infected cells can modulate EV content by inducing the secretion of STING-related factors, to induce antiviral response into neighbor cells (7), or by facilitating miRNA secretion that could control the dissemination of the virus (9).

A growing body of evidence suggests multiple reactivations of HSV-1 as one of the potential risk factors for Alzheimer’s Disease (AD), a neurodegenerative and multifactorial disease that represents the most common form of dementia in the elderly. Indeed, many studies pointed out that subclinical/asymptomatic infection of HSV-1 reaching the brain may produce slight neuronal damage, which, accumulating over time, may predispose the brain to AD (10). We previously showed that several neurotoxic pathways were elicited by HSV-1, such as intra- and extracellular accumulation of amyloid-beta peptide species (Aβ) and their deposition in plaques, hyperphosphorylation/aggregation of the microtubule-associated tau protein (both molecular hallmarks of AD), neuroinflammation, synaptic dysfunction, and impaired adult neurogenesis (11–14).

Interestingly, exo were shown to carry misfolded proteins such as Aβ oligomers and tau protein in AD (15). Higher levels of phosphorylated forms of tau (ptau) have been described in exo isolated from the cerebrospinal fluid (CSF) of AD patients compared with controls (16). Therefore, exo released from brain cells into CSF, but also crossing the brain blood barrier and reaching circulating blood, are becoming a valuable diagnostic tool (17). However, recent findings also support the active role of exo in AD pathogenesis (18). For instance, the spreading of tau through EV can contribute to long-distance dissemination of this protein in the brain thus propagating tau-related pathology. It has been proposed that this misfolded protein could be transmitted trans-synaptically from neuron to neuron in a prion-like manner and act as seeds when captured by recipient cells by initiating intracellular aggregation of endogenous protein (18, 19).

In the present study, we investigated if the HSV-1-induced ptau spreads within the central nervous system (CNS) via EV, thus propagating HSV-1-induced tau-dependent damage within the brain. First, we characterized the tau species secreted in association with EV after HSV-1 infection in neuroblastoma cells or primary neurons, assessing that HSV-1 infection *in vitro* promotes the release of high-molecular weight (MW) tau. Moreover, by overexpressing human tau tagged with GFP (htau^GFP^), we found that EV can transmit HSV-1-induced ptau to recipient neurons. Finally, by exploiting an *in vivo* model of HSV-1 infection, we determined that virus infection reaching the brain upregulates the release of exosomal tau in the brain of infected mice. Our data highlight that HSV-1 could enhance ptau propagation among neuronal cells via EV, thus identifying a new mechanism by which HSV-1 may likely contribute to neurodegeneration.

## RESULTS

### EV-mediated release of phosphorylated tau is upregulated by HSV-1 infection in neuroblastoma cells

**Fig 1:**
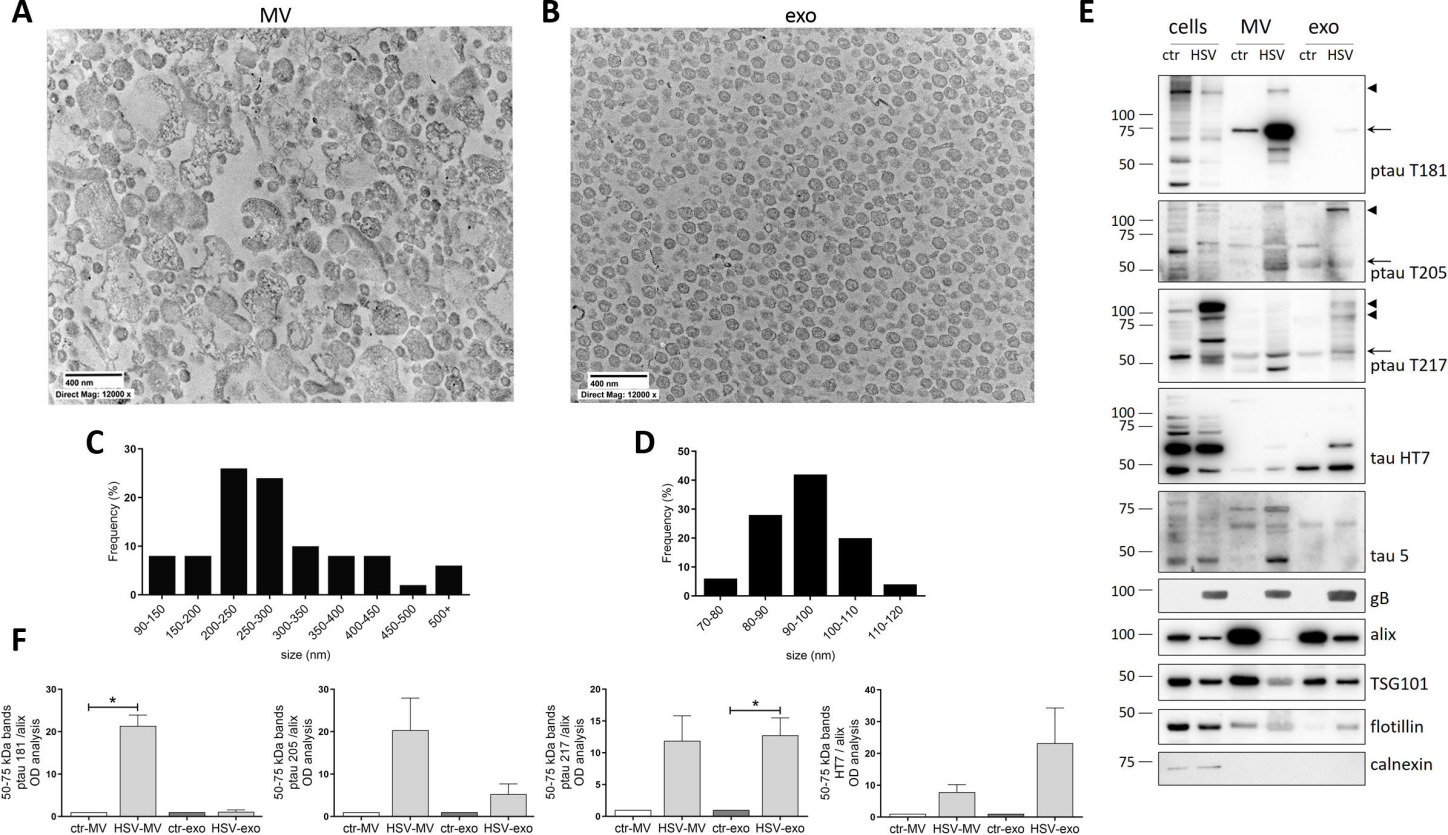
Characterization of EV isolated from SH-SY5Y neuroblastoma cell supernatants upon HSV-1 infection. (**A and B**) Representative TEM micrographs showing the membranous nature of the MV (**A**) and exo (**B**) isolated from supernatants of mock-infected neuroblastoma cultures 36 h p.i. Scale bars are indicated in the pictures. (**C and D**) Size distribution of collected vesicles in MV (**C**) and exo (**D**) samples. Semi-quantitative data were obtained from TEM images measuring the major diameter of *n* = 50 EV. (**E**) Representative immunoblots showing the protein levels for EV markers alix, flotillin, and TSG101; intracellular marker calnexin; tau protein levels; tau phosphorylation sites in T181, T205, and T217 in lysates of cells and EV from mock- (ctr) and HSV-1-infected neuroblastoma cultures harvested 36 h p.i.; long arrows indicate 50–75-kDa tau; arrowheads indicate high-MW tau. (**F**) Densitometric analyses representing the normalized fold-changes of ptau and tau protein levels compared with ctr, performed with ImageLab software and normalized to alix expression in MV and exo. Data are expressed as mean ± standard error of the mean (SEM) of three independent experiments performed in separate days. Statistical significance was calculated using one-sample *t*-test. *P < 0.05 vs ctr.

To evaluate whether HSV-1 infection may promote ptau release via EV, we first infected SH-SY5Y neuroblastoma cells at a multiplicity of infection (moi) of 1 and EV of different sizes (i.e., MV and exo) were isolated from cell supernatants by many differential centrifugation steps following 36 h post-infection (p.i.). As control, EV were isolated from mock-infected cell supernatants under the same experimental conditions. The composition of EV was assessed by transmission electron microscopy (TEM) and western blot (WB) analyses. Images in Fig. 1A and B show the lipidic-membrane-enclosed nature of the isolated particles. As expected, MV samples include vesicles with different sizes whereas exo samples revealed a more homogenous population (Fig. 1C and D; Fig. S1). WB analyses confirmed the purity of the isolation procedure detecting the presence of the EV markers alix, TSG101, and flotillin (1) and the absence of the intracellular marker calnexin (Fig. 1E).

We then checked the presence of tau protein in the cargo of EV (both MV and exo) derived from HSV-1- and mock-infected cells (Fig. 1E and F). We found that HSV-1 infection induced a strong release of different tau isoforms MW ranging from 50 and 75 kDa, indicated in the figure by long arrows) that were phosphorylated at T181 (named hereafter ptau T181), at T205 (ptau T205), and at T217 (ptau T217). In particular, 75-kDa ptau T181 significantly increased in MV, while 50-kDa ptau T205 and T217 were released in both MV and exo (Fig. 1E and F). Regarding total tau protein, we used both HT7 and tau5 antibodies, as their epitopes rely on the mid-domain of human tau. However, in our experience, the H7 antibody allows easier detection of tau protein in human neuroblastoma SH-SY5Y. Indeed, staining with HT7 antibody revealed that a higher amount of protein was present in HSV-EV compared with ctr-EV.

Interestingly, EV isolated from HSV-1 infected cells also contained high-MW ptau (≥100 kDa, phosphorylated at T181 in MV, T205 in MV and exo, and T217 in exo) with respect to EV isolated from mock-infected cells (Fig. 1E, arrowheads), which are compatible with aggregated ptau (20, 21). Overall, our data indicate that HSV-1 infection of human neuroblastoma cells induces the release of ptau pT181, T205, and T217 via EV.

### Following HSV-1 infection, high-MW isoforms of ptau are released from cultured cortical neurons via EV

Next, we checked whether HSV-1 promotes the release of phosphorylated forms of tau via EV also in primary neurons isolated from the cortices of rat embryos, that, differently from SH-SY5Y and SK-N-MC cells (22), recapitulate most of the properties of post-mitotic neurons in the brain. In these cells, we first investigated the profile of tau phosphorylation following HSV-1 infection, since a complete characterization is still lacking.

**Fig 2:**
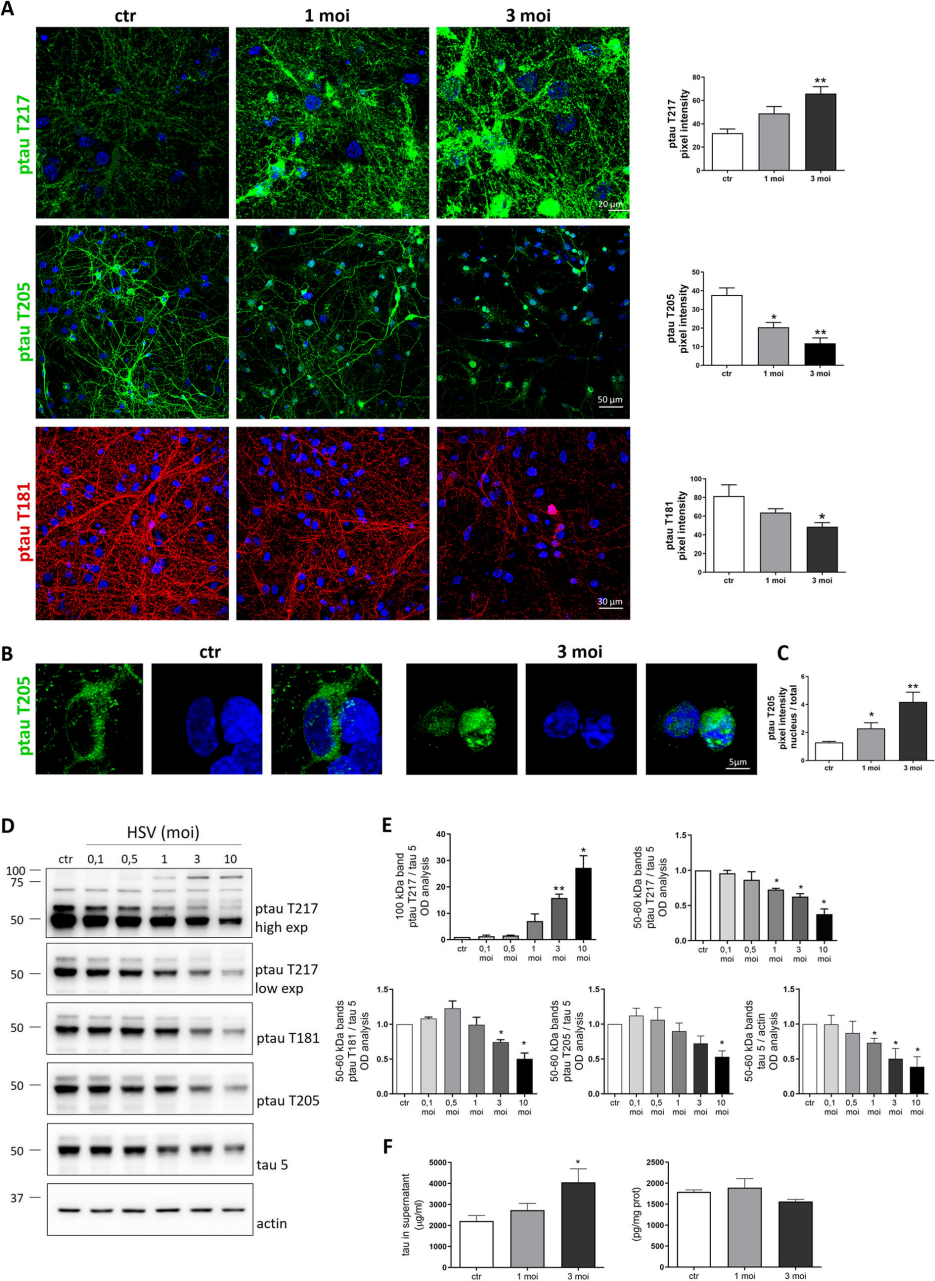
Tau phosphorylation and expression in primary cortical neurons. (**A–C**) Confocal immunofluorescence analyses of primary cortical neurons that were mock- or HSV-1-infected with increasing moi of virus for 24 h. Images in A on the left show representative neurons that were immunostained for ptau T217 or ptau T205 (green) and ptau T181 (red). Magnified images of ptau T205 immunofluorescence are shown in B. Cell nuclei were stained with DAPI (blue). Bar graphs on the right in A show the mean fluorescence intensity of ptau immunoreactive signals. Bar graphs in C show the mean ptau 205 fluorescence intensity measured in the nuclei of all the cells present in the analyzed fields (*n* > 50 cells/field). The intensity was normalized on the total mean fluorescent intensity of ptau T205 measured in the same field. Data are expressed as mean ± SEM and were collected at least from three independent experiments using cultures prepared on separate days. *P < 0.05 and **P < 0.01 vs ctr. Statistical significance was calculated by one-way analysis of variance (ANOVA) followed by Bonferroni post hoc test. (**D**) Representative immunoblots showing tau phosphorylation and protein levels in lysates from mock- (ctr) and HSV-1-infected neurons at the indicated moi harvested 24 h p.i. Actin expression level was used as sample loading control. (**E**) Bar graphs show the densitometric analyses of immunoreactive signals expressed as normalized fold-change of protein compared with ctr. Data are expressed as mean ± SEM of three independent experiments performed in separate days. *P < 0.05 vs ctr. Statistical significance was calculated using one-sample *t*-test. (**F**) ELISA assay for tau was performed on supernatants (left graph) and neuronal lysates (right graphs) collected 24 h after mock and HSV-1 infection. Bar graphs show mean tau protein levels expressed as micrograms (μg) or picograms (pg) of tau protein normalized to milliliters (mL) of supernatant or milligrams (mg) of total protein. Data were collected at least from three independent experiments using cultures prepared on separate days. *P < 0.05 vs ctr. Statistical significance was calculated by one-way ANOVA followed by Bonferroni post hoc test.

We found that rat primary neurons cultured for 14 days *in vitro* (DIV14) and then infected with different moi of HSV-1 exhibited higher immunoreactivity for ptau T217 compared with mock-infected neurons 24 h p.i.: images in Fig. 2A show a ptau T217 immunofluorescence trend increase in a moi-dependent manner that was statistically significant at 3 moi of infection (*P* = 0.0012). Interestingly, WB experiments revealed high-MW bands of ptau T217 (~60 and 90 kDa) that significantly increased compared with controls starting from 3 moi of infection (Fig. 2D and E), whereas low-MW ones (~50–55 kDa) decreased in a dose-response manner. Unexpectedly, we found a decrease of tau phosphorylation in T181 and T205, both by immunofluorescence (Fig. 2A) and by WB analyses (Fig. 2D and E). The latter specifically detected a significant decrease in the levels of 50-kDa phosphorylated bands, as well as in 50-kDa total tau band (Fig. 2D and E) at the higher moi. Notably, the immunofluorescence staining for all ptau forms revealed a localization of the phosphorylated protein that is decentered toward cell nuclei in infected cells (Fig. 2A through C). In particular, confocal analysis revealed that the intensity of the ptau T205 signal in HSV-1-infected neurons was lightly detectable in the neuronal processes and significantly increased in the perinuclear area. This indicates that the majority of the ptau signal was accumulated in the soma in HSV-1-infected neurons (Fig. 2C). Further WB images and immunofluorescence analyses on tau and ptau content in primary neurons that have undergone HSV-1 infection are provided in Fig. S2. Then, we checked whether tau is released from HSV-1-infected neurons by enzyme-linked immunosorbent assay (ELISA). In the same experimental setting, we checked the levels of both the intracellular (i.e., the protein retained in neurons) and the secreted tau (i.e., the protein released in neuron supernatants). Our results in Fig. 2F show that the levels of tau protein retained in HSV-1-infected neurons compared with controls were only slightly decreased (~10%) but were paralleled by a significant increase of total tau in the supernatant. These data indicate that tau could be released from infected neurons in the extracellular medium also in a vesicle-free manner (Fig. 2F).

**Fig 3:**
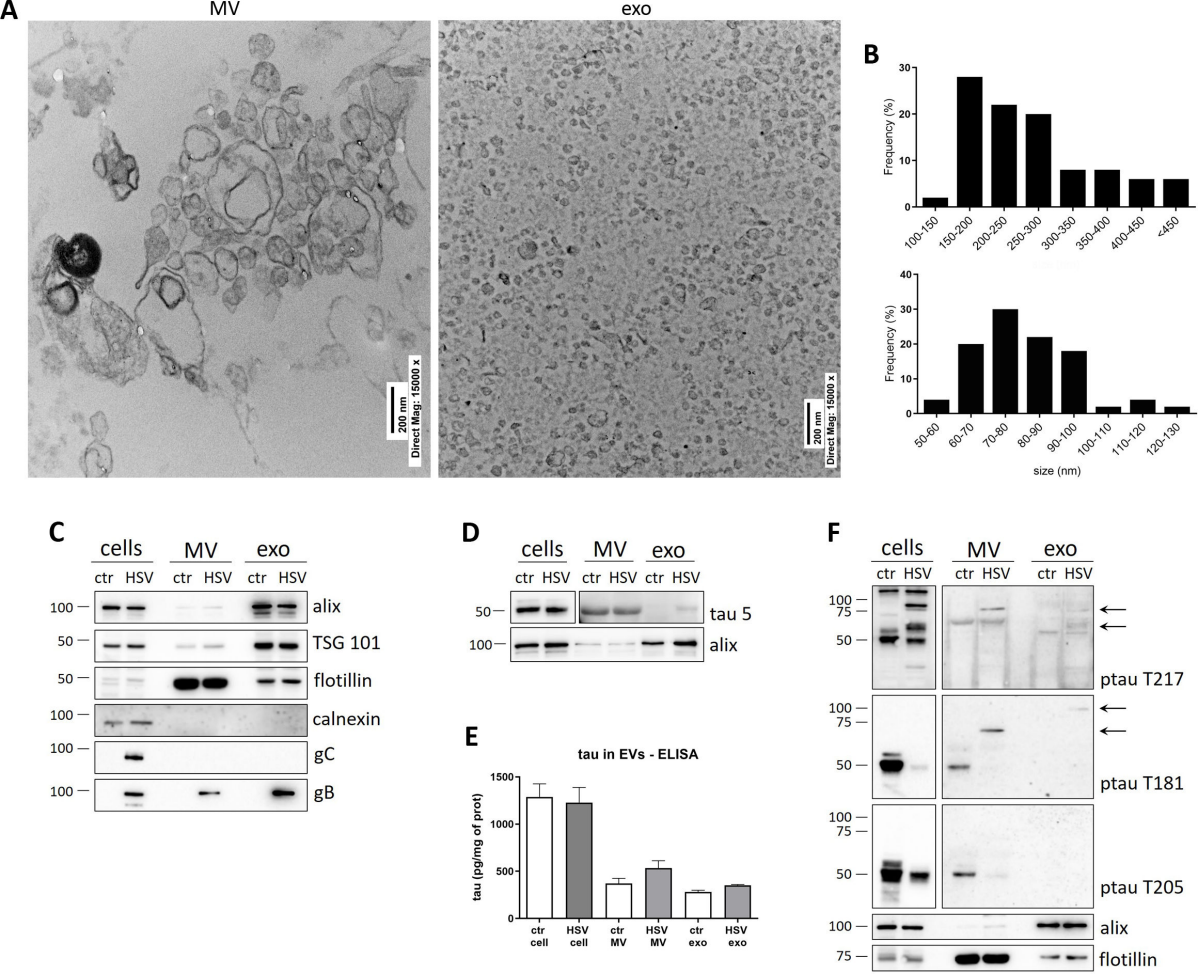
EV purification from primary cortical neurons. (**A**) TEM micrographs showing the membranous nature of the vesicles isolated from supernatants of primary cultures of neurons harvested 36 h p.i. Scale bars are indicated in the pictures. (**B**) Size distribution of collected vesicles in MV (upper graph) and exo (lower graph) samples. Semi-quantitative data were obtained from TEM images measuring the major diameter of *n* = 50 EV. (**C, D, and F**) Representative immunoblots showing the protein levels for (**C**) EV markers alix, flotillin, and TSG101; intracellular marker calnexin; and viral glycoproteins gB and gC; (**D**) tau protein levels; (**F**) tau phosphorylation in the indicated sites, in cells and EV lysates from mock- (ctr) and HSV-1-infected primary cultures harvested 36 h p.i. Blots in D and F represent different exposures of the same membranes: panels on the left show cropped images of lanes loaded with cell lysates (low exposure); panels on the right show cropped images of lanes loaded with MV and exo (high exposure). (**E**) ELISA for tau were performed on neuronal lysates, MV, and exo collected 36 h after HSV-1 infection. Bar graphs show mean tau protein levels expressed as pg of tau protein normalized on mg of total proteins. Data were collected at least from three independent experiments using cultures prepared on separate days. Statistical analysis was carried out by one-way ANOVA followed by Bonferroni post hoc test.

To assess if ptau could be released from neurons via EV, we characterized both MV and exo purified from supernatants of mock- and HSV-1-infected cortical primary neurons harvested 36 h p.i. and evaluated their contents in tau protein isoforms. As we did for neuroblastoma cells (see Fig. 1), TEM analysis allowed us to characterize the size and the morphology of isolated EV (Fig. 3A and B) and WB analyses validated the purity of our EV preparation. These indeed were enriched in EV markers alix, TSG 101, and flotillin but did not show the presence of the intracellular vesicle marker calnexin in comparison to cell lysates (Fig. 3C). As shown in Fig. 3D, tau protein was present both in MV and in exo purified from primary cultured neurons. The levels of total tau cargo in EV and in cell lysates were also quantified by ELISA showing that the amount of the protein in EV did not significantly change during infection (Fig. 3E). Moreover, after infection, both MV and exo contained high-MW isoforms of ptau that were specifically phosphorylated at T217 and T181 (black arrow, right panels, Fig. 3F). Differently from what was observed in neuroblastoma cells, ptau T205 was not found in EV isolated from infected neurons. Our results suggest that HSV-1 infection induces a slight decrease of tau protein retained in the neurons whereas it induces a release of ptau associated with MV and exo.

Collectively, our data suggest that following HSV-1 infection: (i) high-MW isoforms of tau phosphorylated at T217 accumulate in the neurons and are released via EV; (ii) ptau T205 migrates from dendrites to cell soma of infected murine neurons and, accordingly to this re-localization in the nucleus, is not released via EV; (iii) high MW ptau T181 is released via EV by the neurons.

### Exosomal htau released from infected cells is uptaken by primary cortical neurons

It has been reported that EV can transfer tau protein among neuronal cells thus possibly contributing to the spreading of tau pathologies (19). Thus, to assess whether HSV-1 could promote the dissemination of tau via EV, these were purified from murine neuroblastoma cells after HSV-1 or mock infection and layered on recipient neurons of the same species to analyze the possible uptake of EV-bearing tau. For these experiments, we exploited N2A cells as a source of EV and primary culture of neurons from mice that were knocked down for tau (tau KO) as recipient cells to minimize the confounding effects of endogenous tau signal (23, 24). We confirmed the absence of tau expression in tau KO neurons by WB analyses (Fig. S2). N2A were transiently transfected with a vector allowing overexpression of human tau tagged with GFP at the N-terminus (htau^GFP^) to directly monitor the fluorescent protein transmission via EV.

**Fig 4:**
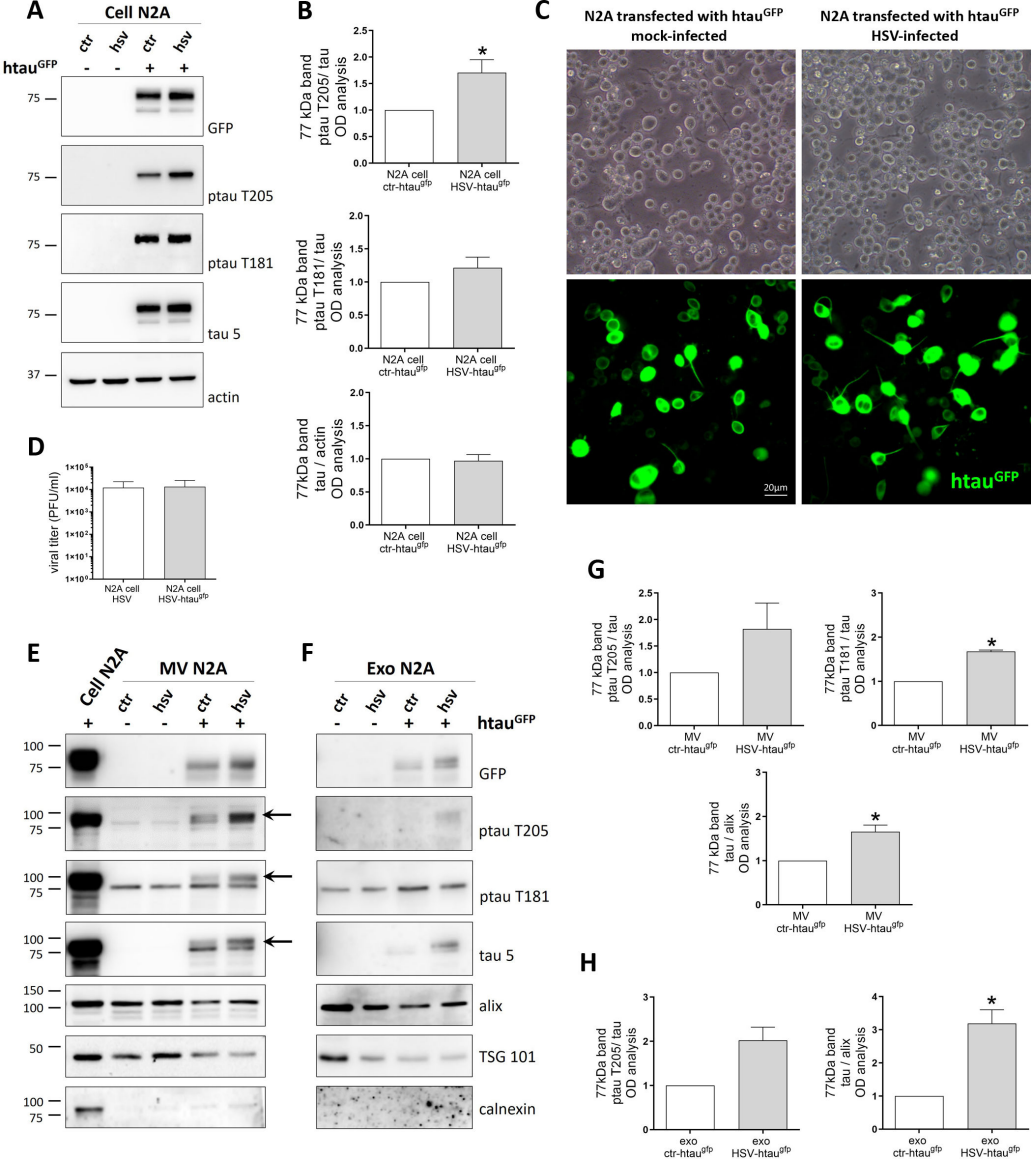
EV purification from N2A-htau^GFP^-transfected cells. Representative immunoblots (**A**), gel densitometry analysis (**B**), and fluorescent images (**C**) showing the overexpression of human tau tagged with GFP (htau^GFP^) in N2A cells. Cells were transfected for 24 h prior to being mock- or HSV-1-infected for 36 h with 1 moi of virus. (**D**) Bar graphs showing HSV-1 titers, calculated by standard plaque assay and expressed as PFU/mL. (**E and F**) Representative immunoblots showing MV (**E**) and exo (**F**) isolated from mock- (ctr) and HSV-1-infected cells. (**G and H**) Bar graphs show the densitometric analyses of immunoreactive signals detected in MV (**G**) and exo (**H**) and expressed as normalized fold-change of protein compared with ctr. Alix expression level was used as sample loading control. Data were collected at least from three independent experiments using cultures prepared on separate days and expressed as mean ± SEM. Statistical significance was calculated using one-sample *t*-test. *P < 0.05 vs ctr.

We first checked whether htau^GFP^ expression/phosphorylation levels were affected by HSV-1 infection. Figure 4A through C shows representative blots, densitometric analyses, and fluorescence microscopy images of mock- and HSV-1-infected N2A cells over-expressing htau^GFP^. As control, the WB analyses in Fig. 4A included native N2A cells (i.e., not-transfected ones), that, as expected, did not show any staining at the MW compatible with htau^GFP^. We found that HSV-1 infection did not modify the expression of htau^GFP^ that migrates under reducing conditions at ~77 kDa (a MW that is compatible with 50-kDa tau + 27-kDa GFP weight) (Fig. 4A and B). Moreover, we found that HSV-1 significantly increased htau^GFP^ phosphorylation at T205 indicating that HSV-1 could modulate htau^GFP^ phosphorylation. We also verified that htau^GFP^ transfection did not alter the efficacy of HSV-1 infection by evaluating viral titer in cell supernatants by standard plaque assay (SPA) (Fig. 4D).

Next, we checked the possible htau^GFP^ release via EV upon HSV-1 infection. As expected, we found that both MV and exo secreted by N2A-transfected cells contained htau^GFP^ (Fig. 4E and F). In addition, the EV from HSV-1-infected cells showed an increased amount of GFP-tagged tau with a different phosphorylation pattern. Specifically, EV-tau was phosphorylated at T181 and T205 in MV (Fig. 4E and G), whereas exo-tau was phosphorylated at T205, but not at T181 (Fig. 4F and H). These results suggest that HSV-1 promotes phosphorylation of human tau and its release into EV.

**Fig 5:**
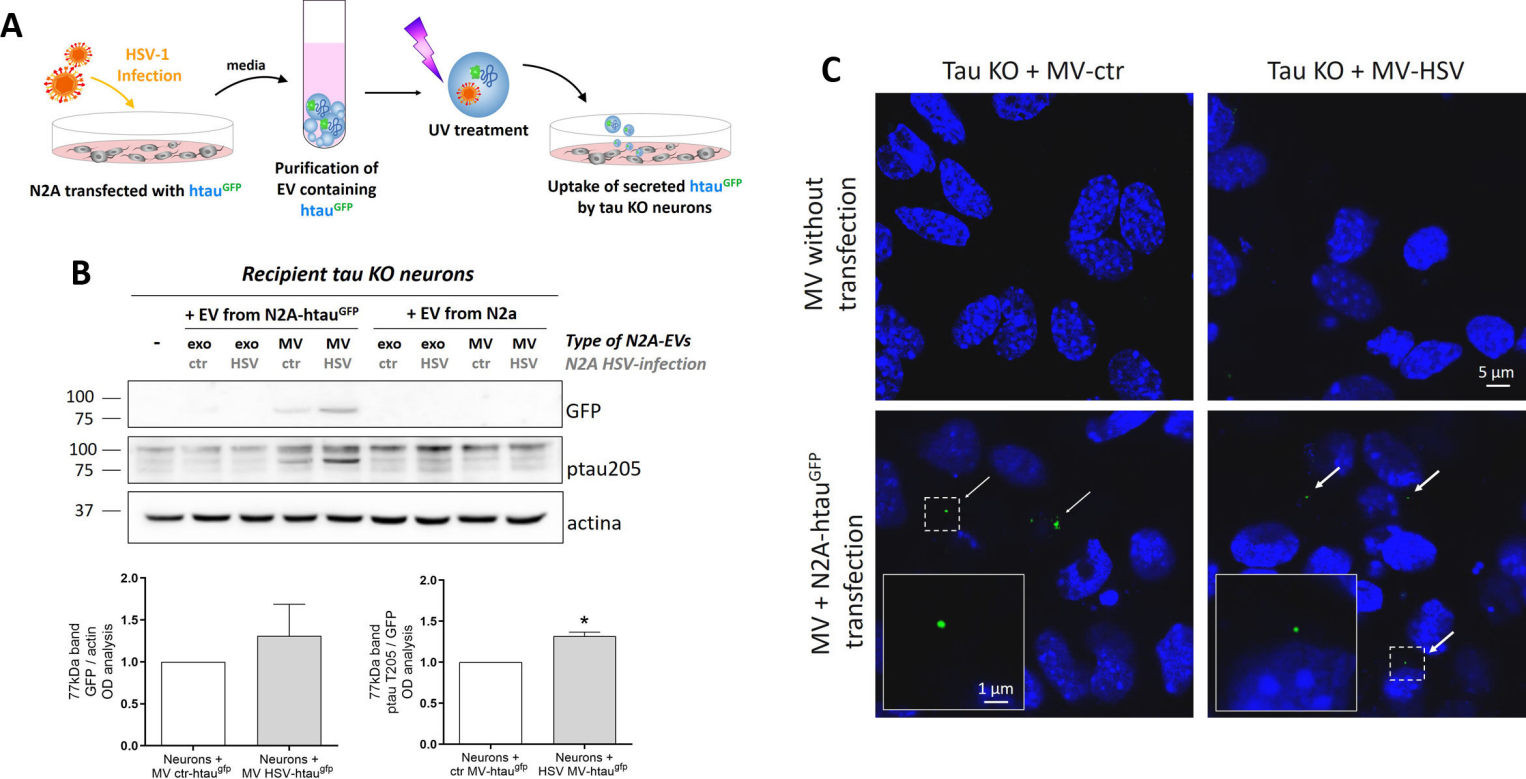
Recipient assay performed with EV purified from htau^GFP^-N2A onto tau KO primary neurons. (**A**) Schematic representation of the experimental procedures for the recipient assay. Mouse neuroblastoma cells (N2A) were transfected for 24 h with an expression vector for htau^GFP^. Following 36 h of mock and HSV-1 infection, EV secreted by transfected cells were collected, treated with UV to inactivate the virus, and layered on tau KO neurons for 24 h. (**B**) Representative high exposure immunoblots showing htau^GFP^ levels and its phosphorylation in T205 in primary KO neuron lysates collected after 24 h of recipient assay (i.e., EV layering). Actin expression level was used as sample loading control. Bar graphs on the right side of the panel show GFP expression normalized to actin and tau phosphorylation at T205 normalized to GFP immunoreactive signal. Data are expressed as mean ± SEM and represented as fold-changes compared with ctr. (**C**) Confocal immunofluorescence images of primary KO neurons that were used as recipients for MV isolated from transfected and non-transfected N2A cells. Arrows indicate GFP^+^ signal. Insets show higher magnification of boxes outlined in each panel. Data were collected at least from three independent experiments using cultures prepared on separate days and were expressed as mean ± SEM. Statistical significance was calculated by one-sample *t*-test. *P < 0.05 vs ctr.

Subsequently, we checked whether EV-tau from HSV-1-infected neuronal cells could be transferred to recipient cells. Thus, we treated purified EV with UV to inactivate the virus likely hidden inside them (5) before layering them onto tau KO-recipient neurons (see schematic representation of the experimental design in Fig. 5A). The same experimental procedure was applied to EV purified from mock-infected N2A-transfected cells containing htau^GFP^. This experimental setting (i.e., tau KO neurons as recipient cells and UV treatment) was required to avoid possible bias in tau expression and phosphorylation due to the endogenous protein and active viral replication in recipient cells. As expected, we did not detect viral replication in recipient neurons under this experimental condition by SPA analyses (data not shown). When we analyzed by WB the lysates from the recipient neurons with the aid of an anti-GFP antibody, we were able to detect htau^GFP^, thus indicating that the EV-htau^GFP^ had been captured by tau KO neurons (Fig. 5B). On the contrary, we could not detect htau^GFP^ in exo. We also checked the effects of UV irradiation on EV morphology and ptau content: results in Fig. S3 indicate that this treatment does not affect EV integrity and tau phosphorylation. Overall, these results indicate that HSV-1 induced the release of tau that can indeed be transmitted among neurons, especially by large EV. Confocal microscopy analyses also confirmed these data since htau^GFP^ were sparsely detected in tau KO neurons, suggesting EV internalizations possibly followed by the release of their fluorescent cargo (Fig. 5C). Moreover, htau^GFP^ that was uptaken by tau KO neurons was phosphorylated only at T205 (Fig. 5B), but not at T181, and the content of ptau 205^GFP^ found inside neurons was increased following HSV-1 infection. Overall, these data provide evidence that HSV-1 could promote the spreading of ptau via EV.

### *In vivo* HSV-1 infection upregulates the release of exosomal tau

**Fig 6:**
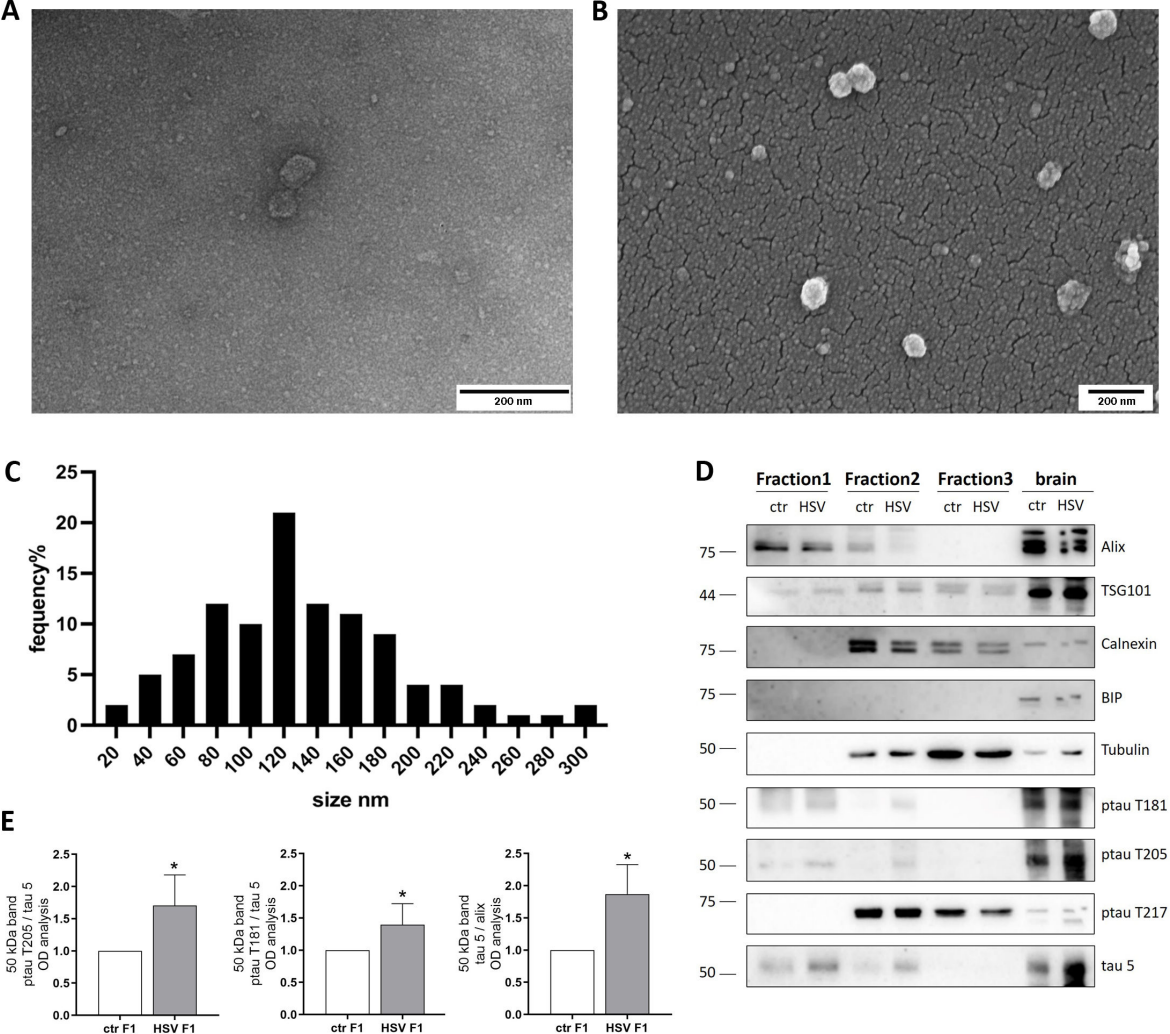
EV purified from *in vivo* mice model of HSV-1 infection. (**A and B**) Representative images of TEM (**A**) and ScEM (**B**) analyses showing the EV isolated from *in vivo* samples. Scale bars are indicated in the figure. (**C**) Size distribution of collected vesicle semi-quantitative data was obtained from TEM images measuring the major diameter of more than 150 EV. (**D**) Representative immunoblots showing tau phosphorylation and protein levels in lysates of different fractions of EV purification from mock- (ctr) and HSV-1-infected mice. Alix expression level was used as sample loading control. (**E**) Densitometric analyses of immunoreactive signals in Fraction 1 (**F1**) are shown in the graphs: values represent the normalized fold-changes in protein levels from HSV-infected mice with respect to ctr (*n* = 5). Data are expressed as mean ± SEM. Statistical significance was calculated by one-sample *t*-test. *P < 0.05 vs ctr.

Based on our findings showing that ptau can be released via EV and uptaken by murine primary neurons, we decided to further gain insight into the spreading of tau within the brain *in vivo*. To this aim, we analyzed brain tissues from a subset of 6-week-old BALB/c mice that we inoculated with HSV-1 or mock solution via lip scarification and sacrificed 4 d.p.i. These mouse tissues were previously virologically and molecularly characterized for the presence of infectious virus (11). EV were isolated from frozen brains as previously described (25) and detailed in Materials and Methods. Compared with *in vitro* experiments, *in vivo* EV isolation requires an additional purification step with sucrose gradient centrifugation to remove debris. EV-enriched fractions were validated by WB, TEM, and scanning electron microscopy (ScEM Fig. 6A and B). The presence of marker alix and TSG101 and the absence of intracellular proteins tubulin, calnexin, and BIP were detected by WB, revealing the fraction of interest as the top one isolated from the gradient (Fraction 1, Fig. 6D). ScEM micrographs of the fraction of interests showed a preparation prevalently consisting of vesicles well dispersed with different sizes (Fig. 6B), while TEM characterization confirmed the lipidic-membrane-enclosed nature of particles (Fig. 6A). Images obtained from EV observation were used to perform a semi-quantitative analysis, which showed non-homogeneous vesicles whose diameters were mainly distributed from 80 to 180 nm with a median value of 121 nm. The analyzed samples also present vesicles with a diameter > 200 and <50 nm (Fig. 6C).

WB analyses revealed that the EV-enriched fractions isolated from the brains of mock- and HSV-infected mice contain tau phosphorylated at T181 and T205 whose levels are increased in HSV-1-infected mice compared with matched controls (Fig. 6D and E). However, we did not find phosphorylation at T217 in the exosomal fraction, but it was present in the other fractions containing intracellular components. Collectively, our results indicate that HSV-1 could enhance ptau propagation within the brain, likely contributing to neurodegeneration.

## DISCUSSION

Data presented in the current study provide novel evidence that EV play a role in the propagation of HSV-1-induced ptau among neurons.

Firstly, we found that the EV released from human neuroblastoma cells post-HSV-1 infection contained increased levels of ptau T205, T217, and T181 compared with EV from mock-infected ones, as well as the total amount of tau, which strongly increased both in MV and in exo (see Fig. 1). These results, in line with previous studies showing the occurrence of tau phosphorylation upon virus infection at T212, S214, AT100 (T212/S214), AT8 (T202/T205), and PHF (S396/S404) (26), highlight for the first time that HSV-1 could drive the phosphorylation of other ptau sites that are also found released via EV.

Noteworthy, we investigated ptau sites that are strongly associated with AD pathology and that are known to be released outside of the cell. In particular, ptau T181 is a well-known marker of early AD (17) since its levels are increased in both CSF-derived exo (16) and neuronal-derived blood EV of AD patients (27, 28). Moreover, other specific ptau forms released in the CSF are commonly used as early markers for AD pathology, and novel ptau sites are currently being investigated (29). Among these, ptau T217 and also ptau T205, which is an already well-known labeling site to define AD progression (30), are emerging as promising CSF markers that could predict the presence of brain amyloid accumulation (29, 31).

Our data also evidenced that, after HSV-1 infection of murine primary neurons, tau protein may be released associated with EV and in a vesicle-free manner (see Fig. 3). The latter may be partly due to HSV-1-induced cell death of infected neurons (32). We focused our interest on the protein that is propagated through EV since previous studies suggested that trans-synaptical exo transmission among neurons play a key role for *in vivo* tau spreading (19, 33). We found that both MV and exo contain high-MW isoforms of ptau T181 and T217. According to our results, we speculate that HSV-1 could induce tau phosphorylation, which, depending on the phosphorylated site, could potentially relocate the tau protein in neurons, such as toward the nucleus or outside the neuron. Indeed, we also noticed that ptau immunoreactivity was mostly decentered toward cell nuclei of primary neurons, especially for ptau T205 (see Fig. 2). These results are in line with published findings reporting a possible link between intranuclear tau and many neurodegenerative diseases (34–36). Specifically, some reports suggested that tau interaction with DNA could possibly either act as a transcript factor or alternatively protect DNA from external damage (37, 38). Along this line, we previously reported that HSV-1 could lead to DNA damage in neurons (39). However, further study will be necessary to clarify whether each HSV-1-induced ptau could be associated to a different fate of the protein.

Our results evidenced some discrepancies between results from human neuroblastoma cells and murine neurons (as the EV cargo of ptau T205). Nevertheless, these could be due to the intrinsic differences between murine and human tau (40) or to the post-mitotic environment of primary cortical neurons, which may deeply impact the outcome of the HSV-1 infection with respect to actively replicating cells. Along this line, further analyses on HSV-1-infected neurons derived from human-induced pluripotent stem cell (41) could fill the gap among these contrasting findings.

It is known that tau protein can be transmitted trans-synaptically following neuronal activity (19), and recent findings support the idea that tau can spread inside the brain, promoting misfolding and aggregation of native tau protein in recipient cells. Indeed, it is well established that exo isolated from CSF of AD patients contained tau seeds that can favor tau aggregation in cultured cells and trigger the neurodegenerative cascade (42, 43). Thus, we investigated whether HSV-1 can exacerbate these pathological tau features, favoring the propagation of aberrant tau isoforms (19). We took advantage of a human tagged tau (htau^GFP^) that we transiently transfected in N2A cells and chose tau KO neurons as recipient cells (Fig. 4 and 5). Our results evidenced that KO neurons captured htau^GFP^ contained in MV derived from infected N2A cells and that it was hyperphosphorylated in the pathogenic site ptau T205 (see Fig. 5). Although we were able to observe the transfer of htau^GFP^ only via MV, we can’t exclude that also exo could transfer a small amount of protein that we could not detect under our experimental conditions. These results provide novel evidence that EV from HSV-1-infected cells could contribute to the dissemination of HSV-1-induced ptau in recipient neurons.

It was previously reported that glycoprotein of other viruses such as vesicular stomatitis virus glycoprotein and spike protein of severe acute respiratory coronavirus 2 could facilitate the spreading of intracellular cargo through ligand-decorated EV (44). We speculate that HSV-1 infection can contribute to EV-mediated tau spreading not only modifying tau phosphorylation profile but also changing the asset of protein inserted in the EV membrane. Indeed, our results indicate that EV derived from infected cells contain a noticeable amount of gB protein (Fig. 1 and 3), which could facilitate the transfer of EV cargo in recipient cells due to its fusogenic features (45).

However, we cannot rule out that a higher amount of EV may be released from infected cells. Indeed, it has been reported that HSV-1 induced a higher release of EV (3); thus, the increase in the captured htau^GFP^ by recipient neurons observed in our experimental model could also be due to the higher amount of EV induced by HSV-1 infection.

Moreover, here, we report that EV purified from the brain of HSV-1-infected mice contained increased levels of tau phosphorylated at T205 with respect to mock-infected ones, confirming that also in our *in vivo* model (11), HSV-1 infection reaching the brain could favor ptau accumulation and its release via EV (Fig. 6). Interestingly, we have previously shown that in our mice, multiple HSV-1 reactivations triggered AD-like hallmarks, including ptau T205 accumulation in the brain (11). According to the results reported here, it is reasonable to hypothesize that this is also due to propagation of ptau T205 via EV. Notably, this result is in line with studies reporting elevated levels of Aβ and ptau (especially in S396 but also in pT181, pS198, pS199, pS202, pT231, and pS404) in EV isolated post-mortem from the brain of AD patients (46). In the present study, we focused on tau protein; however, it is reasonable to hypothesize that EV from infected cells contain other neurotoxic proteins and miRNA that are modulated by the viral infection, such as Aβ and pro-inflammatory cytokines, as well as viral protein and genome, that may affect neighbor cells predisposing them to AD-related neurodegenerative pathways. These issues will deserve further investigation.

Our data also provided novel evidence on the virus-induced tau phosphorylation in primary neurons, which closely recapitulate the characteristics of post-mitotic neurons. We found that within murine neurons, many isoforms of ptau were down-modulated by the virus infection, except for ptau T217 (see Fig. 2). These results are partially consistent with previous papers reporting the occurrence of tau phosphorylation in other sites in primary cultures of neurons following infection with herpesviruses (36, 47, 48). Besides the diverse ptau sites analyzed, the discrepancies between our results and others could be ascribed to differences in the experimental models, e.g., post-natal neurons undergone infection at DIV4 (48) versus our infection protocol on DIV14-neurons, that is when neurons are usually considered mature and tau expression is better established (49, 50). Concerning the overall decrease of tau and ptau, we cannot rule out the possibility that our antibodies did not recognize in WB other aggregated forms of ptau that could be generated following HSV-1 infection. Along this line, our results from ELISA (that likely exploits a different anti-tau antibody) reveal no significant differences in tau levels between controls and HSV-1-infected cells (see Fig. 3). Moreover, previous studies partially analyzed the decrease of total tau expression following HSV-1 infection, but they did not quantify the differences of tau content neither by WB or by IF (47), while the decrease of total tau has been already described following *in vitro* equine virus infection (36).

Different scenarios might explain the role of tau release in EV following herpetic infections. One hypothesis is that HSV-1 could promote tau phosphorylation, which is embedded in EV during virus hijacking exo machinery. According to this hypothesis, the spreading of tau among neuronal cells is a casual downside triggered by herpetic infection, which finally ends with tau aggregation and tangles formation in AD. Another possibility is that HSV-1 may affect cargo protein sorting machinery, modulating proteins that are involved in EV ptau packaging with an unknown mechanism, as ptau was found with a set of specific proteins in EV from familiar AD brains (51). In addition, it has been hypothesized that the viral protein gC could modify the sorting of cellular proteins, such as galectin-1, into EV through a yet-unknown pathway (6). However, another interesting hypothesis that has emerged during the last few years argues that proteins involved in neurodegeneration such as Aβ and tau could act as antimicrobial peptides and restrict virus spreading as part of the CNS innate immune response (52). Along this line, EV from HSV-1-infected cells have been reported to bear both antiviral and proviral factors (53). Further studies are needed to unravel whether tau could have a dual protective/damaging role and to elucidate which viral protein could be involved in tau dissemination.

In conclusion, our data demonstrating that HSV-1 induces the spreading of tau protein among neuronal cells, thus possibly propagating tau-related damage in the brain, further support the role of the virus in neurodegeneration.

## MATERIALS AND METHODS

### Virus production and titration

HSV-1 production (F strain, a wild-type strain, a kind gift from Prof. Manservigi, Ferrara University, Italy) was performed in VERO cells, and the virus preparation was titered by SPA (54) as described in reference (55). Virus titer was 1.7 × 10^8^ PFU/mL.

### Cell culture, *in vitro* infection, transfection, and recipient assay

Neuro-2a (N2A), SH-SY5Y, and VERO (ATCC CRL-2266, CCL-131, and CCL-81, respectively) cells were cultured in appropriate medium supplemented with 10% heat-inactivated fetal bovine serum (FBS), 0.3 mg/mL L-glutamine, 100 U/mL penicillin, and 100 µg/mL streptomycin (Sigma-Aldrich S.r.l., Milan, Italy). Cells were routinely checked for the absence of mycoplasma and other bacterial contamination.

SH-SY5Y cells were seeded at a density of 7 × 10^6^ cells in T175 Flask. After 24 h, cells were mock or HSV-1 infected with 1 moi of virus in serum- and EV-free medium. After adsorption (1 h at 37°C) and phosphate buffered saline (PBS) washes, cells were cultured for 36 h in Dulbecco's Modified Eagle Medium (DMEM) supplemented with 2% EV-free FBS.

N2A cells were plated 48 h before the infection at a density of 3 × 10^5^/well in a six-well plate. The day after, cells (at 70% confluence) were transfected with pRK5-EGFP-Tau plasmid (gifted from Karen Ashe—Addgene plasmid # 46904) (56) using Lipofectamine 3000 (Invitrogen L3000-008), according to the manufacturer’s instruction. Four hours later, cells were supplemented with EV-free FBS. The day after cells were infected with HSV-1 (five moi). After 2 h of incubation at 37°C, the medium was replaced with high-glucose DMEM containing 2% of EV-free FBS. Thirty-six hours p.i., supernatants were collected for exosome purification. For recipient assay, isolated EV were resuspended in Neurobasal and treated for 30’′ under UV light to inactivate HSV-1. UV-treated EV were then layered on DIV 14 tau KO neurons plated in a six-well plate. Twenty-four hours later, cells and supernatants were harvested for further analyses.

Cortical primary neurons were prepared from the brains of E17 WISTAR rat embryos or C57BL/6 tau KO (https://www.jax.org/strain/007251) as previously described (57). Neurons were cultured for 14 days, with half the medium refreshed every 48 h, and then mock or HSV-1 infected with HSV-1 in Neurobasal. After virus incubation (1.5 h at 37°C) and PBS washes, cells returned in the original conditioned media and cultured for 36 h.

Following the infection experiments, cells and supernatants were harvested and processed for the next experiments. Small aliquots of supernatants were exploited to evaluate viral titer by SPA assay as previously described (54).

### EV purification from cell-conditioned medium

EV were isolated 36 h p.i. from cell supernatant by differential centrifugation. For exo isolation, conditioned media derived from 30 × 10^6^ SH-SY5Y, 25 × 10^6^ N2A, or 20 × 10^6^ primary neurons were collected for each condition. The supernatants underwent many centrifugation steps (300 × *g* for 5′, then 1,500 × *g* for 10′, 2,500 × *g* for 15′, and 10,000 × *g* for 30′). The pellet (containing MV) was washed with PBS, centrifuged again at 10,000 × *g*, and then lysed or processed for further experiments. The supernatant resulting from the last centrifugation (containing exo) was ultracentrifuged at 150,000 × *g* for 1.5 h using F40L or F65L rotor in an ultracentrifuge Beckman. The pellet was washed in PBS and lysed in RIPA buffer for WB or ELISA assay or further processed for recipient experiments.

### *In vivo* infections and purification of EV from tissues

We exploited the mouse model of recurrent HSV-1 infection previously set up and virologically characterized (11). Mice were sacrificed 4 days p.i. (4 d.p.i.); brains were collected and immediately frozen in liquid nitrogen. EV were isolated from frozen brain tissue following previously optimized protocols (25). Tissues were weighed and sliced with a scalpel to increase the surface area and incubated with Hibernate-A containing 75 U/mL of collagenase (800 μL/100 mg of tissue) at 37°C for 15′. Tissues were gently pipetted to disaggregate the brains and then further incubated for 5′ at 37°C. To block the enzyme digestion, the tube was then put in ice and protease/phosphatase inhibitor cocktails (Sigma-Aldrich) were added. Brains were then spun at 300 × *g* for 5′ at 4°C, the supernatant was collected and further spun at 2,000 × *g* for 10′ at 4°C and at 10,000 × *g* for 30′ at 4°C and overlaid on a sucrose cushion (0.6M, 1.3M, and 2.5M) that was made by 2 mL of 2.5M sucrose (F4), 1,8 mL of 1.3M sucrose (F3), 1.8 mL of 0.6M sucrose (F2), and 1.8 mL of the supernatant (F1, sample bring to volume with ice-cold PBS). The gradient was spun for 3 h at 180,000 × *g* at 4°C (F65L rotor in Beckman). Fractions were collected and diluted with ice-cold PBS to a final volume of 9 mL. Finally, each fraction was spun at 100,000 × *g* at 4°C for 1.5 h. Pellets were resuspended in 80 µL of PBS and further processed for electron microscopy or lysed in RIPA.

### Western blots

Cells, EV, and brain tissues were homogenized with ice-cold RIPA buffer (20 mM Tris, 150 mM NaCl, 1% Triton X-100, 1% sodium deoxycholate, and 0.1% SDS) supplied with protease and phosphatase inhibitor cocktails (Sigma-Aldrich) and centrifuged at 15,000 × *g* at 4°C for 20′. Protein concentrations were measured with the Micro BCA method (Thermo Fisher Scientific). An equal amount of protein for each sample was separated by SDS-PAGE and blotted onto 0.45-µm nitrocellulose membrane (GE Healthcare) and further processed with the primary and secondary antibodies described in Table 1. The specific density was normalized to β-actin for cells and tissues and to alix protein for EV samples and compared with mock-infected cells or mice.

**TABLE 1:** List of the antibodies used in this study

Antibody	Code	Dilution
Primary		
Actin	Sigma Aldrich A2228	WB 1:5,000
Alix	Invitrogen PA5-52873	WB 1:1,000
Bip	S.cruz 1051	WB 1:2,000
Calnexin	S.cruz 6465	WB 1:1,000
Flotillin	Abcam GR268156-1	WB 1:1,000
gB	cruz 569871	WB 1:1,000
gC	Abcam GR281191-2	WB 1:1,000
gfp	Invitrogen 15256	WB 1:2,000
ptau T181	Invitrogen MN1050	WB 1:1,000 IF 1:200
ptau T205	Abcam ab4841	WB 1:1,000 IF 1:300
ptau S214	Invitrogen 44-742-G	WB 1:500
ptau T217	Invitrogen 44744	WB 1:1,000 IF 1:200
tau 5	Invitrogen MA1-045	WB 1:1,000
tau HT7	Invitrogen MN 1000	WB 1:1,000
TSG 101	PA5 82236	WB 1:1,000
Secondary		
Anti-rabbit HRP	Jackson Immunolab. 711-035-152	WB 1:4,000
Anti-mouse HRP	Jackson Immunolab. 715-035-150	WB 1:5,000
Anti-goat HRP	Jackson Immunolab. 715-035-150	WB 1:5,000
Donkey anti-rabbit 488	Invitrogen A11055	IF 1:700
Donkey anti-mouse 546	Invitrogen A10036	IF 1:700

### Enzyme-linked immunosorbent assay

An ELISA kit (Cusabio, Texas, USA, CSB E13 729) was used to assess total tau levels in primary neurons and EV, according to the manufacturer’s instructions.

### Immunofluorescence

Primary neurons were seeded on 20-mm coverslips that were previously coated with poly-L-lysine. After infection, neurons were fixed with PBS + 4% paraformaldehyde (PFA) for 15′ and permeabilized 10′ at room temperature (RT) with 0.1% Triton X-100 in PBS. Cells were blocked 1 h at RT with PBS + 5% horse serum (HS) and then incubated with primary antibodies diluted PBS + 1% HS. Secondary antibodies coupled to Alexa Fluor dyes were diluted in PBS + 1% HS and incubated 1 h at RT (Thermo Fisher Scientific, Waltham, MA, USA). DAPI was used to stain nuclei. Stained neurons were imaged with a confocal laser scanning microscope (Leica SP5, Leica Microsystems, Wetzlar, Germany) under a sequential mode, to avoid a crosstalk between channels. Images were acquired at 40× or 63× magnification. Image analysis was performed by the ImageJ software (NIH). Quantification of immunofluorescence was carried out by drawing regions of interest (ROIs) and quantifying the mean fluorescence intensity in the ROIs. Quantification of tau immunofluorescence in the nucleus was carried out by drawing a mask on the DAPI channel and quantifying the mean fluorescence intensity of tau within the applied mask.

For production of figures, processing was done by using the CorelDRAW software.

### ScEM and TEM

For ScEM analysis, purified exo were left to adhere to polylysine-treated round glass coverslips (10 mm) for 5 h at RT. According to Shively and Miller (58), samples were first fixed with glutaraldehyde 2.5% in sodium cacodylate buffer and then post-fixed with osmium tetroxide 1%. Samples were dehydrated through a graded series of ethanol and hexamethyldisilazane solutions and then final drying and leaving to evaporate for 2 h (58). Dried coverslips were mounted on stubs, gold sputtered (10 nm), and analyzed by GeminiSEM 450 (Carl Zeiss).

EV isolated from cells were fixed overnight (ON) at 4°C in 2% glutaraldehyde in 0.1 M phosphate buffer pH 7.3. After rinsing in the same buffer, samples were post-fixed in 1.33% osmium tetroxide in the same buffer for 2 h at room temperature. Samples were dehydrated in increasing ethanol concentrations, passed in toluene, and embedded in Epon resin. After polymerization overnight at 65°C, 80-nm sections were cut on a Leica UC7 ultramicrotome and picked up on copper grids. Sections were stained in UranyLess and then lead hydroxide. The sections were observed in a Jeol 1400-plus TEM.

TEM analysis of small EV derived from brain tissues was performed by a negative staining method (59). Ten microliters of samples was deposited on carbon-coated grids for 5′, and grids were air-dried. A mix of phosphotungstic acid 2% and ammonium molybdate 4% (pH 6.8) (1:1 ratio) were added on grids for contrast. TEM samples were examined at 100 kV by PHILIPS EM208S TEM (FEI—Thermo Fisher Scientific), equipped with the Megaview II SIS camera (Olympus). A semiquantitative analysis was performed by measuring diameters of at least 150 vesicles per sample using open-source software Fiji (60).

### Statistical analysis

Statistical comparisons were performed with GraphPad 6.0 (Prism). Data are presented as mean ± SEM. All analyses were two tailed and *P* values < 0.05 were considered statistically significant. To assess the effect of HSV-1 infection on protein content in EV or neurons, fold-changes of infected samples were compared with matched controls by one-sample *t*-test. To analyze ptau intensity and total tau levels by ELISA assay, means were compared by one-way ANOVA followed by the Bonferroni post hoc test.

## Data Availability

All relevant data supporting the findings of this study are available within the manuscript and its Supplementary materials.

## SUPPLEMENTAL MATERIAL

Supplemental figures mbio.01522-24-s0001.pdf Fig. S1 to S3.

ASM does not own the copyrights to Supplemental Material that may be linked to, or accessed through, an article. The authors have granted ASM a non-exclusive, world-wide license to publish the Supplemental Material files. Please contact the corresponding author directly for reuse.
