# Supplementary material for: HSV-1 infection induces phosphorylated tau propagation among neurons via extracellular vesicles
Source: mBio. 2024 Aug 27;15(10):e01522-24. doi: 10.1128/mbio.01522-24 (PMC11481531; doi:10.1128/mbio.01522-24)
Supplement: Supplemental figures — Fig. S1 to S3. [file mbio.01522-24-s0001.pdf]

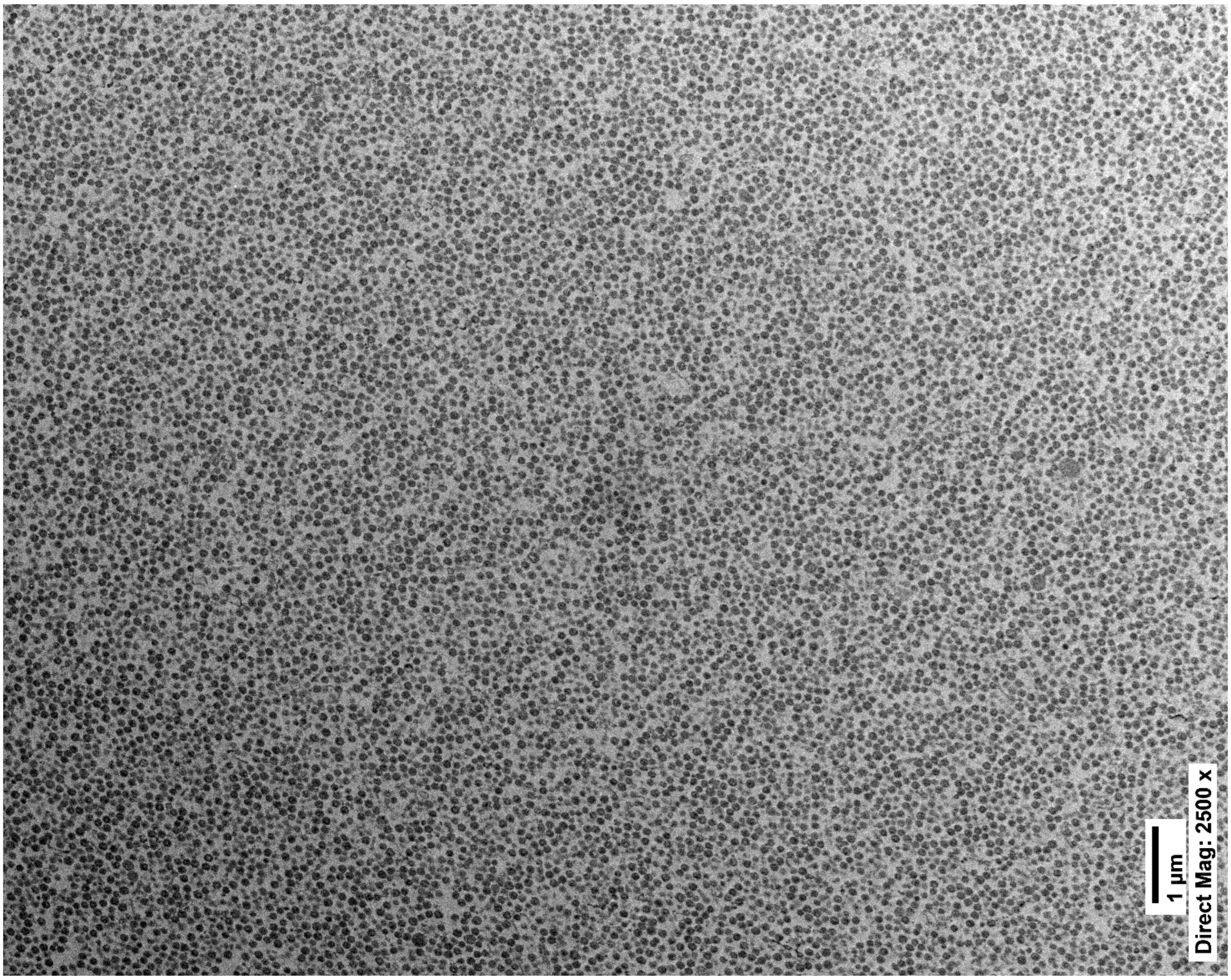

Fig supplementary 1. Exosome population of SH-SY5Y Representative TEM micrographs showing exo isolated from supernatants of mock-infected neuroblastoma cultures 36h post infection (p.i.) at low magnification, showing the homogenous nature of the isolated vesicles. Scale bars are indicated in the picture

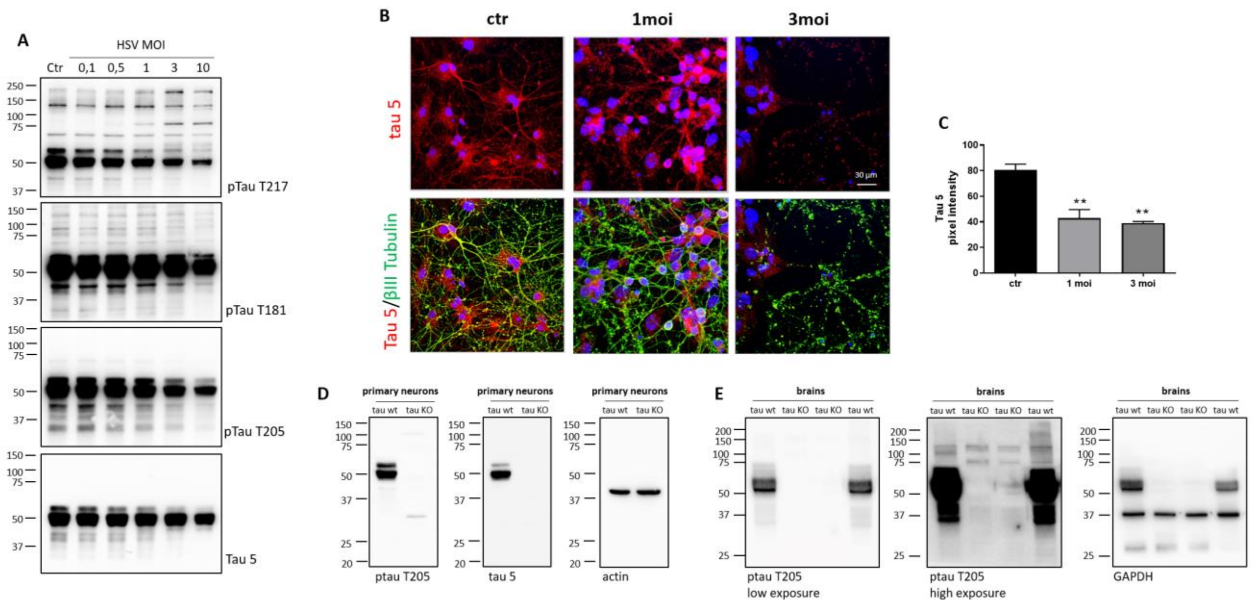

Fig supplementary 2. Supplementary information about tau and ptau expression in primary neurons. A) Complete immunoblots at high exposure of gels showed in Fig 2D. B-C) Confocal immunofluorescence analysis of primary cortical neurons that were mock- or HSV-1-infected with increasing moi of virus for 24h. Representative images in B) show neurons that were immunostained for total tau (detected with tau 5 antibody, red) and with βIII tubulin (green). Cell nuclei were stained with DAPI (blue). Bar graphs in C) show the mean tau fluorescence intensity and are expressed as mean ± SEM. Data were collected at least from three independent experiments using cultures prepared on separate days. Statistical significance was calculated by one-way ANOVA followed by Bonferroni post-hoc test, *$p < 0.05$, **$p < 0.01$ vs ctr. D-E) Representative immunoblots showing the expression of tau and ptau T205 in wild type (tau wt) and tau KO primary neurons (D) and brains (E). Actin and GAPDH expression levels was used as sample loading control.

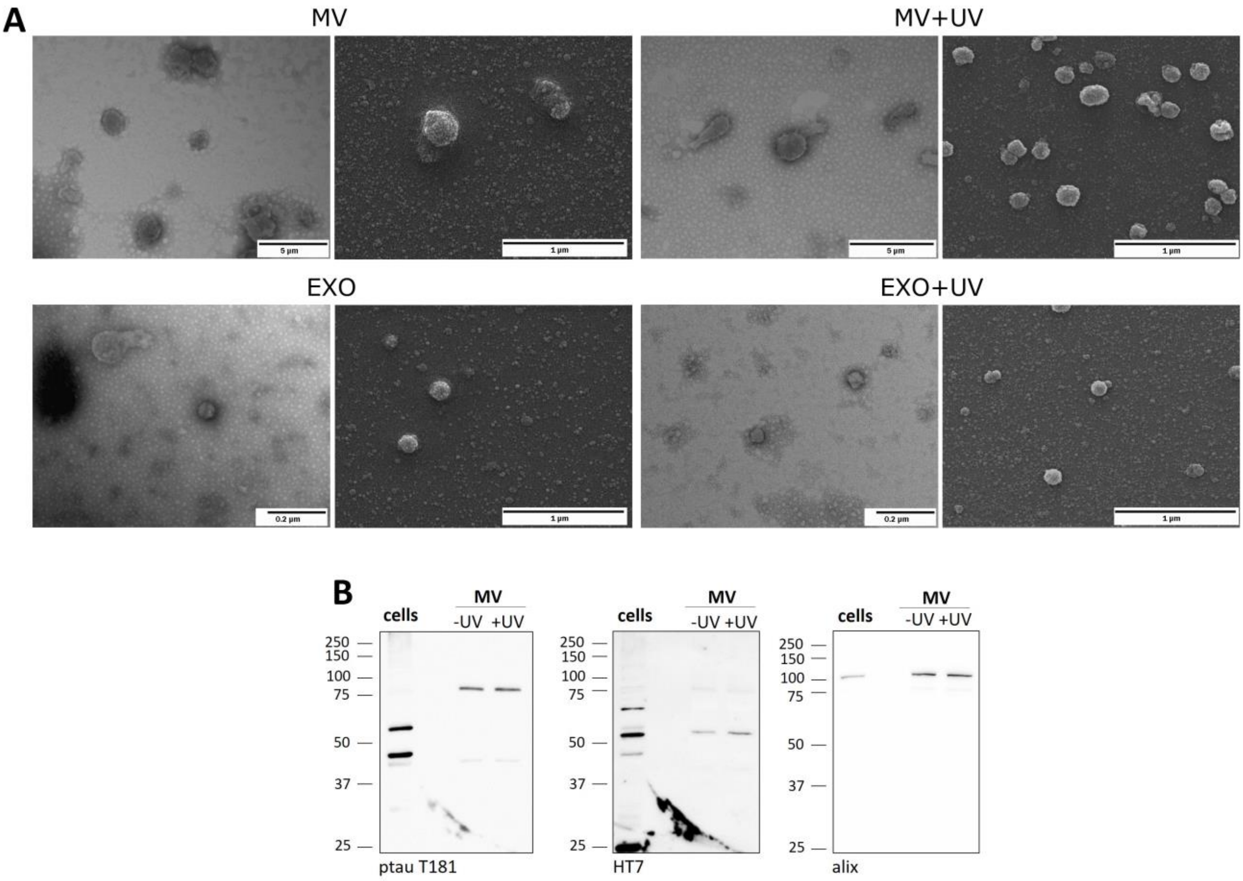

Fig supplementary 3. UV exposure does not affect EV integrity and tau phosphorylation A) Representative TEM micrographs showing MV and exo isolated from supernatants of mock-infected neuroblastoma cultures and then treated or not with UV rays for 30 min. Scale bars are indicated in the picture. B) Complete immunoblots of MV isolated from neuroblastoma cells and treated as in A), showing the expression of ptau T181 and total tau (revealed by HT7 antibody). Alix expression levels was used as sample loading control.
